# Select Clinical Reports; with Observations

**Published:** 1845-01

**Authors:** 


### Select Clinical Reports; with Observations. By *George Barlow*, M.D. (Guy's Hospital Reports, October, 1844.)

This is one of the most important papers in the October number of "Guy's Hospital Reports," and reflects the highest credit on Dr. Barlow, as a man of philosophic mind, accurate observation, and excellent judgement. Such reports as these will stimulate the medical officers of our great establishments to select the more intelligent of their pupils to record with fidelity, the interesting cases that daily occur in the routine of their practice, and communicate them to the less fortunate of their brethren, for their guidance in the private walks of life, and thus diffuse the benefits of their experience among all ranks of society. Such communications, even in a mercenary point of view, are the best passports to fortune as well as fame, and the medical officers of our public institutions would find their fee-books greatly increased in size by their suffering their brethren to participate in the inestimable opportunities which they, themselves, enjoy.

The philanthropic spirit of the Sainted Guy (for he richly deserves *Sainthood*) seems to have descended on the mighty building which he erected for suffering humanity, and to animate all its conductors with the zeal of Christian charity. They have set a noble example to other hospitals, metropolitan and provincial, in the publication of their Reports, which will transmit their names to posterity as disinterested benefactors to the human race, by their efforts to contribute to the stock of practical knowledge.

What is SAINT THOMAS about, in the immediate vicinity of ST. GUY? Is he reading Homilies instead of recording histories and cases? Is SAINT BARTHOLOMEW more engaged in the study of zoology outside of his walls than of pathology within his gates? Is SAINT GEORGE, in the aristocratic West-end, still tilting at the dragon—staring at the great green man in the Park—or lost in admiration of the splendid corteges that move down to Buckingham Palace? What are the grave and learned DOCTORES ET CHIRURGI, of that castellated mansion that faces the venerable Cathedral in Westminster, doing? Are the mephitic vapours that issue from the old cemetery, near Lincoln's-inn-fields, benumbing the activity of our worthy brethren in that quarter? Why do not the great Provincials open the records of their medical and chirurgical transactions, and allow the treasures of their experience to circulate on the four winds? Why have our able contemporaries of ERIN, slackened their pace, and ceased to carry out the good work which they so auspiciously and ably commenced a few years ago, in the shape of the "DUBLIN HOSPITAL REPORTS?" We trust that a new era is approaching, when the example of "GUY'S" will dispel the cimmerian cloud that has, too long, hovered over institutions designed for the benefit of all, and not for the gain of a few!

But we must retûrn to Dr. Barlow. The machinery of the clinical establishment at Guy's deserves praise and imitation. We recommend it to those who are thinking of following the same path.

The first subject that occupies the attention of our talented author is CONSTIPATION of the bowels, illustrated by cases and remarks. It fills more than forty pages of the Reports.

### CONSTIPATION.

It is natural and prudent when a case of serious constipation presents itself, to ask ourselves where is the seat of the obstruction—and what is its nature and cause? These questions are more easily asked than answered. Various symptoms, indeed, indicative of danger, are laid down by systematic authors—as sickness, pain, tenderness and tumefaction of abdomen—high-coloured urine, &c. but none of these are invariably present—some of them absent, even in fatal cases. The fact is, that accurate diagnosis is difficult—and even the most experienced of us are prone to error.

*Case* 1.—J. J. aged 26 years, was admitted on the 8th of March, 1844. Had had an attack of constipation twelve years previously, which yielded to aperients in a few days. Eleven years subsequently he had a similar attack lasting six days, and removed by the long tube. Eight days before admission he had an evacuation, after which he was constipated, with a screwing pain in the epigastrium. On admission, the countenance was anxious—tongue moist—pulse small, 85—abdomen much distended by flatus—track of colon resonant on percussion—no sickness—urinates freely. Five grains of calomel and one of opium statim—castor oil two hours afterwards—warm bath, which gave temporary relief to pain—assafœtida glysters returned, with very little fæculent matter. 9th. Much the same—bath repeated, and whilst there, the flexible tube, to the extent of 18 inches, was introduced, passing through fæcal matter, and giving vent to some flatus. No action of bowels. Four grains of colocynth with one of hyosciamus, every hour. 10th. No relief being obtained, six grains of calomel with one of opium. The long tube again, to 23 inches, and a quart of warm water injected. Some air and slightly tinged water returned—solution of five grains of tartarized antimony in three ounces of water thrown into the rectum. 11th. The pain recurring in paroxysms—warm-bath—warm water copiously injected—returned merely tinged. Twelve grains of calomel followed by castor-oil, repeated in three hours. Same evening, the pain and distention increased—countenance more anxious—pulse full and quick—tongue furred. Tube again introduced 23 inches—large discharge of flatus, and, after injection of warm water, a discharge of green fæcal matter, followed by softness of abdomen and disposition to sleep. 12th. Re-accumulation of air—no more fæces. Nine grains of Barbadoes aloes every three hours—abdomen rubbed with croton oil—tube introduced in the evening, followed by discharge of fluid fæces and air—turpentine enema. 13th, evening. Dusky countenance—jactitation—much pain in the loins—great distention—nausea—hiccup. One pound of crude mercury exhibited, with cessation of hiccup and nausea, and less distention—no evacuation. 14th. Dr. Addison in consultation. Venesection to syncope, with a minim and a half of croton oil. Blood firmly coagulated, but not buffed—much improved after bleeding—stomach irritated by the croton oil—passed some fragments of fæcal matter with much straining—green fæcal fluid drawn off by the tube—a grain of calomel with one of opium every four hours. 15th. The bowels washed out by the tube—discharge of green fluid fæces. Colocynth and hyosciamus pills every hour for six hours—turpentine enema in the evening—calomel and opium at bed-time.

16th. Much the same, and in consultation with Mr. Cooper, as to an operation, it was determined to wait another day, before opening the colon from the loins. Calomel and opium every three hours—larger discharge than hitherto of fæcal matter by the tube.

17th. Passed this morning, two pints of dirty green fæcal matter, well tinged with bile. He seemed better for a time, but gradually sunk on the 19th, with symptoms of ruptured bowel.

*Sectio Cadaveris, 20th March, 16 hours after death.*—"On reflecting the integuments of the abdomen, its cavity was found to be almost filled by an enormously-distended, thickened, and hypertrophic sigmoid flexure, which had been twice turned on its axis. The descending colon, where it became continuous with this distended sigmoid flexure, passed in front of the rectum. Just above this portion of the colon there was ulceration of the mucous membrane, and in one place perforation of the intestine. The sigmoid colon had a twisted root, with a contracted mesentery; it crossed to the right, and suddenly back again: the upper part seemed the largest, but the whole resembled the stomach of a great turtle with a thickened and inflamed mesentery. The rectum was wide and pale; but immediately above the part where the intestine passed behind the descending colon, dilatation with thickening rapidly commenced. There was great muscular hypertrophy of the sigmoid flexure, and a firm, thick lining, in part diphtheritic. There were no ulcers in this part. The transverse and descending colon were much dilated, tumid, and inflamed, diphtheritic and sloughing, with numerous ulcers: one of them had perforated the intestine just above the commencement of the sigmoid flexure. The small intestines were full and inflamed, and feebly glued together in parts with whitish soft fibrine: there was, besides, extensive recent peritonitis.

"The liver, spleen, and kidneys were healthy.

"The left colon was, after death, easily opened through the abdominal walls from behind." 375.

In the course of a long and practical life, we have met with many cases of the above kind—some fatal, some fortunate. For many years past, we have made up our minds to this point, that, whenever constipation exceeds two or three days, inflammation and its various consequences—adhesions, ulceration, perforation, &c. are to be dreaded, and the grand objects of prevention. How fatally inflammation worked its way, and induced its devastations in the above case, a glance at the *post-mortem* must convince the least experienced reader! We confess, therefore, that we should have adopted the suggestion of Dr. Addison at a much earlier period than when that talented physician was called into consultation. We have no doubt that, in the case above described, certain mechanical elements of obstruction existed long before he came into hospital; but we submit that these very circumstances rendered it doubly necessary that depletory measures should be early adopted. Even at the late period when they were employed, a great, though not permanent, amelioration of the symptoms followed the venesection.

We have several times exhibited quicksilver in such cases, but never once with success; and as to the operation of penetrating the colon, from behind, it is perfectly evident that it would have been of no use in the above melancholy case. Dr. Barlow makes many judicious remarks on the subject of constipation generally, and on that just related in particular. His candour in the narration of cases is above all praise, and the value of such candour can be appreciated only by those who have lived long enough in the world to know the care which is taken to keep in oblivion all cases that do not puff off the skill and the good fortune of the narrator.

*Case* 2.—Joseph H——, aged 12 years, admitted 30th March, 1844, employed as a servant at a zinc and scuttle-maker's, Newington, for four months past. Had had an attack of colic and constipation, fourteen months previously, which was soon relieved by purgatives. Some similar attacks occurred afterwards. On the 24th March, was suddenly seized with severe pain in the abdomen, succeeded by violent retching, which continued all night, and next day Mr. Otway saw him, and prescribed various remedies, without effect. For six days before entering the hospital he passed neither fæces nor urine. The surface was now cold and pallid—face congested—expression anxious—eyes sunken—and the whole aspect indicative of serious mischief. There was tendency to stupor—voice feeble —tongue clean and dry—no blue line on the gums—no great distention of abdomen nor tenderness on pressure—no urine for five days (it was stated six days just before)—he sunk and died on the 1st of April.

*Post-mortem, 24 hours after death.*—" *Chest.* The lungs were rather watery, and somewhat mottled with red spots and very slight fleshiness.

" *Abdomen.*—On the surface of the peritoneum were scanty fibrinous deposit, redness, and discolouration, with slight cellular adhesions. The stomach was dilated with gas, and contained dark bilious fluid. About four turns of the upper part of the jejunum were widely distended, dark, and rather thickened: the three lower of them the most sound. At the end of this portion was a distinct, thick, soft, circular cord, of rather recent cellular membrane, obstructing the gut, but adhering to it loosely at most parts; a large probe passed easily under the cord: the beginning of the chief dilatation in the duodenum was also bound to it, as also an intermediate portion. When laid open, the bowel seemed remarkably free. The whole contained fluid fæces. Liver rather tumid. Kidneys full and soft. Pancreas red." 380.

The rationale of this case appears to be, what Dr. Barlow suggests—old peritonitis—frequent subsequent attacks—adhesions—impediment to peristaltic motion and passages of fæces—false membranes constricting the canal, and ultimately giving rise to fatal obstruction and death.

In this case, also, we trace the destructive ravages of inflammation, series after series, till death closed the scene! When the patient was brought into Hospital, nothing, of course, could be of any use, or even procrastinate his fate; but is it not evident that, in all the preceding attacks, especially the earlier ones, depletion might, in all probability, have entirely prevented the series of evils that ensued?

The two cases just related, presented a wide difference in their features—probably from a difference in the chief seats of their maladies.

" 1. In the first case there was little or no abdominal pain at the commencement: in the second it was one of the earliest symptoms.

" 2. In the first case there was no sickness till a very late period: in the second it was urgent from the beginning.

" 3. In the first case there was great flatulent distention of the abdomen: in the second there was none.

" 4. In the first the urine was abundant in quantity: in the second there was an almost entire suppression of that secretion.

" 5. In the first case the circulation was well maintained in the extremities, until the symptoms of perforation manifested themselves: in the second there was early collapse." 381.

Dr. Barlow follows up these contrasts by a series of admirable as well as ingenious reflections on the causes of the discrepancies in the symptoms. We strongly recommend his remarks to the attention of every practitioner. One passage, however, we are tempted to quote.

" The contrast between these two cases in the quantity of the urinary secretion is so remarkable, as almost at once to lead to the belief that it was the necessary result of the difference in the seat or nature of the obstruction; a belief which is strengthened by the fact, that in Case 2, the suppression of urine came on at the same time with the other symptoms. And, further, in cases of this nature, where no fluids can pass into the alimentary canal, and where, in consequence, none can be absorbed, we should be led to expect, *à priori*, that the urine would be very little in quantity. We have, then, rational grounds for believing, that, in cases of constipation where the urine is very deficient in quantity, the obstruction is probably at the upper part of the canal; and that where it is abundant, at the lower.*" 383.

A number of most judicious hints as to the treatment of ileus are given by Dr. B. in which he appreciates the value of blood-letting. But, alas! the time for this measure is too generally gone by before patients enter hospitals. The necessity for attending to this important point, however, by private practitioners, is not the less obvious.

*Case* 3.—This is a more cheering one than either of the preceding. Augustus B——, aged 32 years, was admitted on the 3rd of April, 1844, by trade a tailor, and subject to relaxed bowels, and they had not become confined till six days before admission—during which time he had thrown up everything from his stomach.

*Symptoms on admission.*—Thirst, debility, anorexia—fever—depression of spirits—tongue highly coated—chest resonant—irritable action of heart—constipation of six days—urine loaded with the lithates—abdomen not distended, yet tympanitic—little pain. Pills of Barbadoes aloes every three hours—no effect from the pills—a turpentine enema, with very slight effect. Colocynth, calomel and hyosciamus, followed ultimately by a turpentine enema, brought away a copious motion, his bowels afterwards acting spontaneously, put an end to the complaint.

With the following interesting quotation we shall wind up Dr. Barlow's excellent report.

"In the preceding observations considerable stress has been laid upon the quantity of the urinary secretion, as a diagnostic sign of the situation of the obstruction in cases of constipation: for it seems that where there existed a perfect obstruction in the upper part of the small intestines, there was almost a total suppression of urine; where there was a diminution of the calibre of the canal in the same situation the urine was diminished in quantity; and where the small intestines were free, and the obstruction was seated in the colon, the urine was very abundant. If, then, we regard this statement of facts merely in a diagnostic point of view, as aiding us in determining the probable seat of obstruction in the alimentary canal, it will not be without its use; for every symptom must now be considered important which can help us to decide a question, the answer to which will go far towards determining the expediency of endeavouring to relieve an insurmountable obstruction by surgical operation.

"It is not, however, merely in regard to this diagnostic significance in this or that disease that we are to regard a circumstance that meets us under so many different, and even opposite conditions of the system; when the skin is dry and parched, and when there is excessive perspiration;—in profuse diarrhœa, and in insurmountable constipation;—in disease of the arteries, as well as of the veins;—of the lungs, as well as of the heart;—of the liver, as well as of the kidneys. The occurrence of this single point of resemblance in conditions of the system often diametrically opposite, is a fact too remarkable to be passed over, and is one, the further investigation of which may possibly throw some light upon the circumstances which determine the quantity of the urinary secretion, and afford some assistance in directing our efforts to regulate it."

"The sources whence the urine is derived are now, I believe, acknowledged

---

* "Where the obstruction is low down in the small intestines there will be no suppression."

by most physiologists to be three. 1. The water taken into the alimentary canal with the ingesta. 2. Those soluble substances which are taken in the same way, and which do not undergo decomposition in passing through the extreme circulation of, *e. g.* the neutral salts of metallic bases with mineral acids, which appear to act as stimulants to the kidneys. 3. Those lower organic products which are formed in the extreme circulation, or, in the phraseology of Liebig, the products of the transformation of tissues, *e. g. urea, urates, &c.* In order, then, to the due secretion of urine, the following conditions are essential:—An absorption of a sufficient quantity of water from the ingesta to hold the salts, the decomposed soluble matters, and the products of the transformation of tissues, in solution: the free transit of this water and soluble matters through the portal veins, the liver, the right side of the heart, the lungs, and the left heart, to the kidneys: a healthy condition of the extreme circulation, whereby the normal product of the transformation of tissues may be duly formed: a condition of the kidneys adequate to the carrying all these matters out of the system when applied to them by the blood. If, therefore, any one of these conditions be vitiated by disease, the secretion of urine will be impeded, either as regards its quantity or the proportion of the solid contents." 397.

Appended to Dr. Barlow's paper is a short case communicated by Dr. Lever.

Mrs. P——, aged 51, presented herself to Dr. L. with symptoms resembling the passage of gall-stones—excessive pain in the epigastrium, in paroxysms—jaundice—sickness—scanty urine—constipation, &c. &c. *Treatment.* Local depletion—calomel and opium—castor oil—enemata. The symptoms were removed, with the exception of the yellow tinge—and twelve months elapsed, with constipation and indigestion.

*On the 5th of August,* Dr. Lever was called to her, and found her labouring under excessive pain in the region of the gall-bladder—sickness incessant—extreme yellow tinge—bowels confined—urine high-coloured and scanty. Local depletion—calomel and opium—enemata, &c. But she suddenly sunk and died. The liver was found large and dark-coloured—no trace of gall-bladder—adhesions between the duodeno-pyloric orifice of stomach, and liver and pancreas. Pyloric orifice very much contracted, from thickening of coats. About the middle of the duodenum a common quill could scarcely be passed through. Great contraction also obtained in the transverse descending colon and rectum, which would scarcely admit the point of a finger. About the centre of the ilium there was a biliary calculus the size of a walnut, partially sacculated.

